# Endoscopic ultrasound-guided partial splenic embolization for hypersplenism: a novel alternative

**DOI:** 10.1055/a-2244-4009

**Published:** 2024-02-15

**Authors:** Ping Liu, Shan-shan Zhu, Xin-Guang Cao, Pradermchai Kongkam, Saif Ullah, Chang-Qing Guo

**Affiliations:** 1 191599 Gastroenterology, The First Affiliated Hospital of Zhengzhou University, Zhengzhou, China; 2 26683 Division of Hospital and Ambulatory Medicine and Division of Gastroenterology, Department of Internal Medicine, Faculty of Medicine, and Pancreas Research Unit, Chulalongkorn University, Bangkok, Thailand

Variceal bleeding and hypersplenism are two major complications of portal hypertension, for which the management includes partial splenic embolization (PSE) 1 . A recent multicenter clinical trial confirmed that the combination of endoscopy and radiography-guided partial splenic embolization (X-PSE) provided advantages over conventional endoscopic treatment alone for the prevention of variceal rebleeding in patients with liver cirrhosis 2 . X-PSE requires arterial puncture via the femoral artery and relies on radiographic imaging to guide the catheter, with risks including post-embolization syndrome. In addition, the method is not suitable for patients who are unwilling or unsuitable to undergo radiation, such as pregnant patients or patients planning to conceive.

Anatomically, the splenic artery is near to the gastric wall and is easily visualized by endoscopic ultrasound (EUS). Transgastric puncture of the splenic artery provides the shortest surgical route and does not require radiographic assistance, which led Chen and colleagues to propose endoscopic ultrasound-guided partial splenic embolization (EUS-PSE); they also conducted preliminary research into the prevention of variceal bleeding 2 3 . Recently, we successfully performed EUS-PSE for the treatment of hypersplenism. Here we share our insights with fellow colleagues who might be considering similar procedures.

A 50-year-old woman with decompensated liver cirrhosis of cryptogenic origin was admitted to our hospital with hypersplenism. Laboratory tests and imaging studies, including computed tomography (CT), were consistent with the diagnosis. Esophagogastroduodenoscopy showed small esophageal varices, without gastric fundal varices. After explaining the standard of care and discussing the alternatives with the patient and her family, we decided to proceed to EUS-PSE.

First, the celiac trunk's abdominal branch was visually traced to the splenic artery at the splenic hilum using pulsed wave Doppler ( Fig. 1 ). EUS-guided puncture of a splenic artery close to the splenic hilum was then performed using a 19G EUS-specific puncture needle, and a coil (Nester Embolization Coil MWCE-35-14-6-NESTER; Cook Medical, Winston-Salem, North Carolina, USA) was placed ( Fig. 2 **a** ). Subsequently, polyvinyl alcohol particles, tissue adhesive, and polyvinyl alcohol were sequentially injected. Immediately post-procedure, observation revealed the disappearance of blood flow signals in the target splenic artery ( Fig. 2 **b** ; Video 1 ). The patient did not experience any abdominal pain, bloating, or vomiting, which would have been suggestive of post-embolization syndrome. No other complications, such as pleural or abdominal effusion, splenic or portal vein thrombosis, or splenic abscess, occurred. At follow-up examination 1 month later, her platelet count had increased from 54 × 10 ^9^ /L pre-procedure to 115 × 10 ^9^ /L. An enhanced CT scan showed the coil was positioned at the splenic hilum, with no ectopic embolization, and approximately one-third of the spleen had been embolized ( Fig. 3 ).

**Fig. 1:**
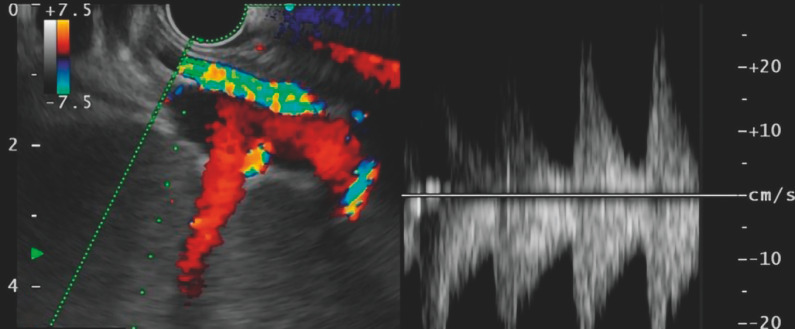
Pulsed wave Doppler image showing the peak blood flow signal at the splenic artery into the splenic hilum.

**Fig. 2:**
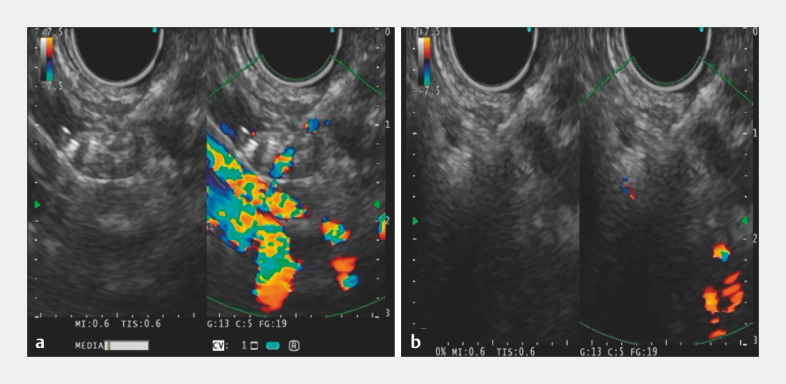
Endoscopic ultrasound (EUS) images showing: **a** EUS-guided placement of a spring coil in the splenic artery; **b** disappearance of the target splenic artery blood flow signal immediately after the procedure.

Endoscopic ultrasound-guided partial splenic embolization (EUS-PSE) is performed in a patient with hypersplenism.Video 1

**Fig. 3:**
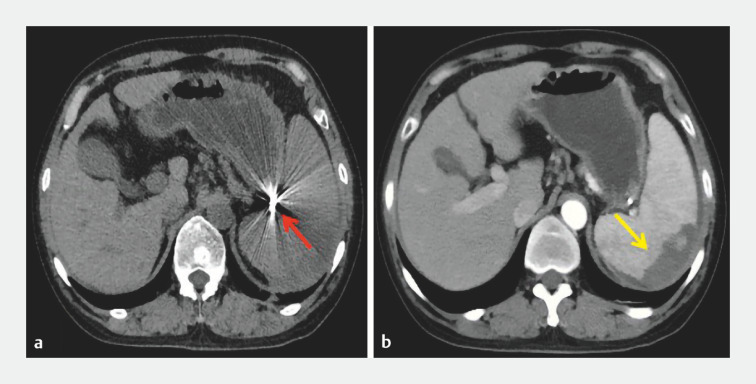
Enhanced computed tomography images showing: **a** the spring coil located at the splenic hilum (red arrow); **b** embolization of about one-third of the spleen (yellow arrow).

In contrast to the traditional X-PSE treatment approach, the patient did not experience abdominal pain and bloating associated with the post-embolization syndrome and had no complications such as splenic abscess or splenic vein thrombosis. The advantages of the endoscopic approach include not requiring radiation exposure or guidewire assistance. The surgical route was short, as was the procedure time, while allowing precise embolization, reduced costs, and a short hospital stay. We propose EUS-PSE as an effective alternative endoscopic treatment for decompensated liver cirrhosis complicated by hypersplenism.

Endoscopy_UCTN_Code_TTT_1AS_2AG

## Notes

Endoscopy E-Videos https://eref.thieme.de/e-videos
*E-Videos* is an open access online section of the journal *Endoscopy* , reporting on interesting cases and new techniques in gastroenterological endoscopy. All papers include a high-quality video and are published with a Creative Commons CC-BY license. Endoscopy E-Videos qualify for HINARI discounts and waivers and eligibility is automatically checked during the submission process. We grant 100% waivers to articles whose corresponding authors are based in Group A countries and 50% waivers to those who are based in Group B countries as classified by Research4Life (see: https://www.research4life.org/access/eligibility/ ). This section has its own submission website at https://mc.manuscriptcentral.com/e-videos .

## References

[1] Zhang ZG Li Z Yang Y Hemodynamic effect through a novel endoscopic intervention in management of varices and hypersplenism (with video) Gastrointest Endosc 2022 95 172 18300 34224735 10.1016/j.gie.2021.06.029

[2] Sun X Zhang A Zhou T Partial splenic embolization combined with endoscopic therapies and NSBB decreases the variceal rebleeding rate in cirrhosis patients with hypersplenism: a multicenter randomized controlled trial Hepatol Int 2021 15 741 752 33638769 10.1007/s12072-021-10155-0 PMC8286949

[3] Chen Q Li Z Yang Y Partial splenic embolization through endoscopic ultrasound-guided implantation of coil as a potential technique to treat portal hypertension Endoscopy 2021 53 E40 E41 32483779 10.1055/a-1174-5590

